# Online Monitoring of Moisture Diffusion in Carbon Fiber Composites Using Miniaturized Flexible Material Integrated Sensors

**DOI:** 10.3390/s19081748

**Published:** 2019-04-12

**Authors:** Martina Hübner, Dennis Lepke, Elisabeth Hardi, Michael Koerdt, Axel S. Herrmann, Walter Lang

**Affiliations:** 1 University Bremen, Institute for Microsensors, -actuators and -systems (IMSAS), Otto-Hahn-Allee 1, 28215 Bremen, Germany; dlepke@uni-bremen.de (D.L.); wlang@imsas.uni-bremen.de (W.L.); 2 Faserinstitut Bremen e.V., Am Biologischen Garten 2, 28359 Bremen, Germany; hardi@faserinstitut.de (E.H.); mkoerdt@faserinstitut.de (M.K.); herrmann@faserinstitut.de (A.S.H.)

**Keywords:** Moisture diffusion, CFRP, interdigital miniaturized sensor

## Abstract

Moisture diffusion in carbon fiber composites changes the mechanical properties of the composite. Therefore, a monitoring method of the actual content of moisture in the composite is important. However, at the moment there are no online methods established. A common method is the measurement of the mass changes due to water uptake. This method is not suitable for online monitoring of a real composite part in service. We demonstrate that miniaturized flexible interdigital sensors are suitable for moisture measurement inside the carbon fiber composite. These sensors are directly integrated inside the composite. It was already demonstrated that these can be successfully used for resin-curing monitoring as a primary application. Here we demonstrate that the same sensors are also suitable for moisture measurement inside the material. In order to do so, we expose samples with and without integrated sensors to hot-wet conditions and measure the dielectric changes with the sensors and the mass gain. The moisture concentration and the measured admittance can be directly correlated to each other. This demonstrates that the sensors can be used for moisture measurement as a secondary application. In addition, it is shown that the sensors have the potential to measure the moisture locally inside the material.

## 1. Introduction

Carbon fiber-reinforced polymers (CFRP) have an increased range of applications due to their properties of being lightweight and having a high stiffness at the same time. In addition, they offer great freedom in the design. Therefore, they are used in industries like automotive, aerospace and marine. In all these areas, CFRPs are exposed to different levels of moisture, which can diffuse in the composite. The fibers absorb nearly no water compared to the matrix material, therefore the main influence of the moisture diffusion is related to the matrix material [1]. This influences the structural properties of the CFRP component due to two different reasons. First, the glass transition temperature T_g_ is lowered [2]. The water diffusion in the polymer matrix leads to the interruption of the van der Waals bonds and decreases the stiffness of the matrix [3]. The second important effect is the hygroscopic swelling of the matrix [4]. This result in internal stress of the composite because the matrix swells while the swelling of the fibers may be neglected since these absorb a much smaller amount of water than the matrix. During the fabrication process, a certain amount of moisture inside the composite may be considered and tolerated in the later CFRP component. Hence, it is important to measure the moisture content in a CFRP component to monitor if it is still in the acceptable range or already degrading its mechanical properties. Several studies demonstrated that the mechanical properties of the CFRP were significantly influenced by the moisture uptake [1,2,5,6,7,8,9]

To evaluate the effect of different environmental conditions on a specific CFRP, samples are exposed to a humid environment [6,10], warm water or seawater [5,7]. The mass of the samples is measured over time. From the mass changes, the amount of moisture inside the CRFP can be calculated. However, this standard weight method can be used to evaluate the general moisture-related behaviour but not to measure the humidity in the CFRP component during service time. In the aerospace sector, so-called “traveler specimens” are used [11]. They are attached to the aeroplane and, in this way, exposed to the same environmental changes than a specific aeroplane part. They can be detached and a measurement can be performed. A test with such a sample showed that up to one percent of moisture was absorbed over several years. However, only a few measurements were taken since with this method no real-time monitoring is possible. Changes between the measurements could not be considered. 

Krauklis proposed the near-infrared spectroscopy as a method superior to the gravimetric analysis [12]. However, this method can also not be used online during the service time of the product. Chinquin showed that Lamb waves are sensitive to moisture uptake in carbon fiber composites [13]. This specific type of surface waves was created and measured with external sensors. The disadvantage of this method is, that Lamb waves are also sensitive to mechanical changes and damages which are not related to the humidity uptake [14,15]. Davis demonstrated that electrochemical impedance spectroscopy is also suitable to measure the moisture content at a CFRP to concrete bond [16]. In this study, electrodes glued on the surface of the CFRP were used. A similar method was described by Shimamura [17]. However, this method is highly dependent on the CFRP part itself. For complex shapes and layups, the results may be difficult to interpret. In addition, external electrodes may not be tolerated. Therefore, sensors should be integrated into the material. 

Salas evaluated different types of humidity sensors that could be integrated into composite materials [18]. In order to integrate sensors in composite materials, the sensors have to fulfill several criteria. First of all, they have to withstand the embedding procedure, considering the chemical reactions, temperature and pressure, without losing their functionality. Second, they should not disturb the fabrication process itself. Due to these criteria, a commercial resistive and a commercial capacitive sensor were chosen and the moisture was successfully measured. However, both types of sensors where quite large and therefore it can be expected that they will disrupt the structural mechanics of the CFRP part [19]. Hence, they cannot be used for online monitoring during the service life of a composite component. 

We presented a miniaturized flexible interdigital sensor for online cure monitoring of carbon fiber composites [20,21,22]. This sensor is designed in such a way that it does not harm the mechanical properties of the composite component [23]. Consequently, it can remain inside the part during its whole service life. To further benefit from this already embedded sensor, it can be used for structural health monitoring. Interdigital sensors, in general, are also used for humidity sensing. Ali presented a printed interdigital structure with a top layer of graphene and methyl-red [24]. This layer absorbs the moisture and results in changes in the dielectric parameters of the layer, which is measured by the interdigital structure. The same concept is also realized with different polymer layers, like polyvinyl alcohol and lithium chloride [25], poly methyl-methacrylate and cellulose acetate [26], polypyrrole [27] or with porous materials like silicon [28] or MnWO_4_ [29]. Martin demonstrated successful measurement of moisture in glass fiber composite with a humidity sensor consisting of nanoporous silica particles embedded in a polyvinyl butyral matrix sandwiched between two layers of electrodes [30]. This sensor had a thickness of at least 50 µm and dimensions in the range of several of centimeters. This indicates that the sensor will probably influence the mechanical properties of the fiber composite. Several problems occur when using this kind of sensors to measure the moisture inside the carbon fiber composite compared to glass fiber. The first is that the sensors have to withstand the embedding procedure. If they are still working after the embedding procedure, these sensors do not measure directly the amount of water inside the CFRP but the amount of water in a specific layer, which is part of the sensor. The values only correspond when the CFRP and the layer which is measured are in equilibrium. 

Here, we demonstrate that the miniaturized interdigital sensors integrated into the fiber composite can measure the moisture uptake of the CFRP directly without an additional medium. We use our flexible sensor that was already used for cure monitoring and can, therefore, be used for multiple applications, which is quite cost-effective. First of all, we briefly describe the production process of the sensor and the CRFP samples. Then, the moisture measurement along with the theory is described. From the weight gain measurements of the samples, we approximate the diffusion coefficient. The dielectric measurements performed with the material integrated sensors are compared to the weight gain curves. It is demonstrated that these can be correlated with each other. The integrated sensor is, therefore, suitable to measure the moisture uptake of the CFRP part. 

## 2. Materials and Methods

### 2.1. Sensor Fabrication 

**Figure 1:**
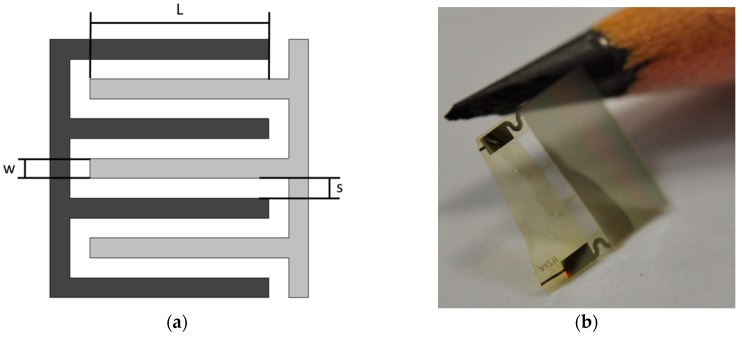
(**a**) Schematic of the interdigital sensor. (**b**) Photograph of the sensor compared to a pencil tip. The sensor area is 3 mm × 6 mm.

The used sensor consists of an interdigital structure on a flexible substrate. A schematic of the sensor is shown in Figure 1a. The geometrical parameters of the used sensors are shown in Table 1, where N is the number of fingers. The electrodes are fabricated on a 5 µm thick polyimide (PI) foil. To further reduce the influence of the sensor on the structural properties of the CFRP, holes are created in the substrate. This results in a net-shaped substrate with net structure dimensions in the range of the diameter of the fiber [21]. The sensor is fabricated in the cleanroom using microfabrication techniques. In the first step, a polyimide (PI) precursor (UPIA–ST from UBE) is spin coated on a silicon wafer, which only acts as a handling device here. The polyimide is cured on a vacuum hotplate with a temperature profile specified by the manufacturer. It results in a 5 µm thick PI layer. On this PI layer, a 300 nm gold layer is sputtered and wet chemically structured in a photolithographic process. On top of the electrodes, a second PI layer is created to have electrical isolation. This top isolation layer has a thickness of around 500 nm. The sensors are lifted from the wafer and wired. For the wiring, copper wires of 127 µm diameter with PI coating are used. The connection of the wires and sensors is realized with conductive silver glue. The contacted area is isolated afterwards. In Figure 1b a photograph of the sensor is shown. 

### 2.2. Composite Fabrication

**Figure 2:**
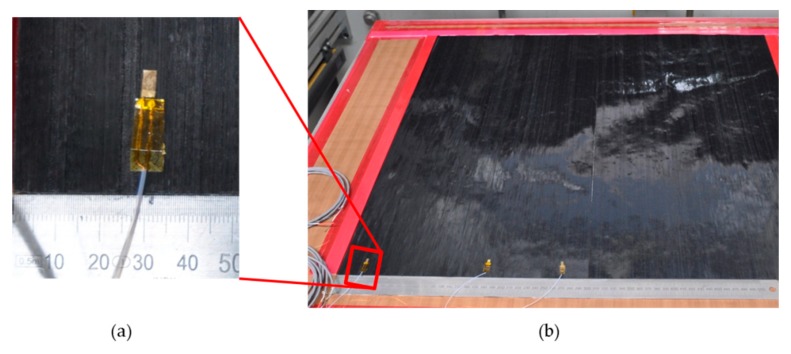
(**a**) Sensor during the integration in carbon fiber composite. (**b**)Carbon fiber prepreg plate with sensors during fabrication.

**Figure 3:**
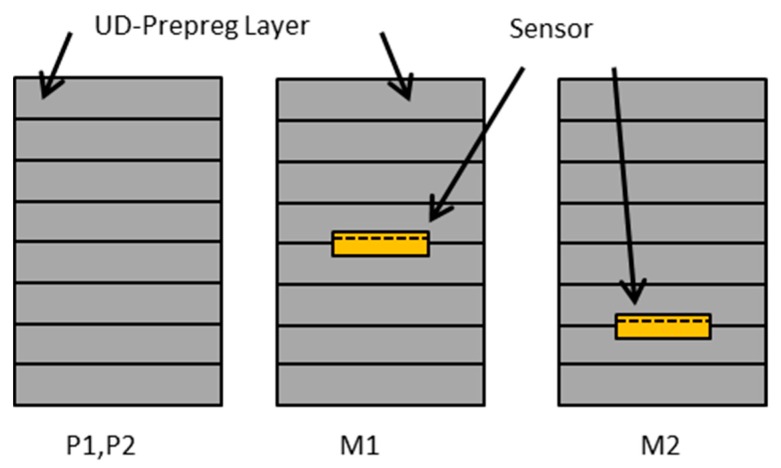
Schematic of the different samples uses. The dimensions are not in scale. The thickness of the prepreg layers is around 2 mm while the sensor thickness is approximately 6 µm. The fibers are oriented unidirectionally. The fibers direction is along the out of plane axis.

The CFRP samples are fabricated from unidirectional (UD) prepreg material. The matrix material is HexPly M21(Hexel) and the fibers are of type T800S (Torayca) (Hexply M21-34_268T800S-ATL300LAU). The matrix resin is an epoxy which is specially designed for aerospace applications. It is designed to cure at 180 °C. Eight layers of prepreg are used and placed in a unidirectional orientation. In between the layers, the sensors can be placed. Here the sensors are placed between layers 2 and 3, and layers 4 and 5. In Figure 2 the embedding of the sensors in the laminate is shown. The laminate is stacked between two aluminium plates. Between these plates and surrounding the laminate, aluminium strips with a thickness of 2 mm are placed as spacers. The whole setup is placed in a vacuum bag to improve the quality of the composite plate by reducing the amount of air bubbles inside. The setup including the vacuum bag is placed in a press with two heating plates. The press is closed and the temperature is set to 180°C. During the whole fabrication process, the sensors are connected to an impedance measurement tool and the impedance changes are recorded. Therefore, the point of minimum viscosity can be measured [20,21,31]. When this point is reached, approximately after 30 min, the pressure of the press is slightly increased to a maximum pressure of 8 bar. Temperature and pressure are released after 4 h. The whole setup remains in the press until it is cooled down. Afterwards, the CRFP plate is demolded. The plate is sawed in different samples with and without sensors. The dimensions of the samples are shown in Table 2 where b is the width and d is the length. All samples have a thickness of 2 mm. In Figure 3, a schematic of the stacking of the different samples is shown. The samples P1 and P2 include no sensor. In sample M1 the sensor S1 is located between layers 4 and 5, which results in a distance of 1 mm from each side. Sample M2 includes the sensor S2 between layers 2 and 3. This sensor has a distance of roughly 0.5 mm from the bottom of the sample (backside of the sensor) and 1.5 mm from the other surface of the CFRP (top side of the sensor). 

### 2.3. Moisture Measurements

The CFRP samples from the plate with and without sensors are dried in an oven at 70 °C for 14 h. Afterwards, they are placed in a climate chamber, which is set at 70 °C and 85 % relative humidity. The samples without sensors are used to determine the weight increase, while the samples with sensors are continuously connected to the impedance measurement tool. For the impedance measurement, an IVIUM n-stat device was used. The impedance is measured automatically in a range from 1 Hz to 1 kHz in 10 logarithmic distributed steps per decade to get a broad information set. The time distance between two measurements starts with 1 min and it is increased during the whole measurement time to several hours due to the fast changes at the beginning of the diffusion process and the slow changes at the time saturation is reached. 

The mass changes are measured by removing the samples P1 and P2 from the climate chamber and measuring the weight with a scale. The scale has a precision of 0.01 mg, while the measured changes are around one mg. In the first days of the experiments, this procedure is repeated several times a day. The frequency of the weight measurements decreases due to the theoretically expected saturation, see the following section. 

### 2.4. Theory of Moisture Diffusion

Diffusion in isotropic materials like polymers can be described by Fick’s law. Diffusion in CFRP can be described in a similar way. Here the diffusion coefficients in the different directions are not equal anymore. However previous publications have shown that Fick’s laws can also be applied to the diffusion in CFRP [3,32,33]. Assuming that no diffusion along the edge happens, Fick’s first law can be reduced to one dimension [32]

(1) $$\dot{m}=-D\frac{\partial c}{\partial z}$$

where $\dot{m}$ is the amount of mass passing the area of the plate, D is the diffusion coefficient, c is the moisture concentration and z the direction of diffusion through the plate. Fick’s second law for the one dimensional case is given as

(2) $$\frac{\partial c}{\partial \tau }=D\frac{\partial^{2}c}{\partial z^{2}}\;.$$

In case of diffusion from two sides, a solution is given as [32]

(3) $$\frac{c-c_{i}}{c_{m}-c_{i}}=1-\frac{4}{\pi }\overset{\infty }{\underset{j=0}{\sum }}\frac{1}{\left(2j+1\right)}\sin\frac{\left(2j+1\right)\pi z}{d}\exp\left[-\frac{{\left(2j+1\right)}^{2}\pi^{2}Dt}{d^{2}}\right]$$

where $c_{i}$ is the initial concentration inside the plate, $c_{m}$ is the maximum saturation concentration, d is the thickness of the plate and D the diffusion coefficient. This equation describes the moisture concentration at every place at every time inside the plate. To compare the theoretical value to the measured mass changes, Equation (3) is integrated over time

(4) $$\frac{m-m_{i}}{m_{m}-m_{i}}=1-\frac{8}{\pi^{2}}\overset{\infty }{\underset{j=1}{\sum }}\frac{1}{{\left(2j+1\right)}^{2}}\exp\left[-\frac{{\left(2j+1\right)}^{2}\pi^{2}Dt}{d^{2}}\right]$$

with $m_{i}$ initial mass of moisture and $m_{m}$ mass of moisture at saturation time. An approximate form of Equation (4) is given by Shen [32]

(5) $$\frac{m\left(t\right)}{m_{m}}\approx 1-\exp\left[-7.3{\left(\frac{Dt}{d^{2}}\right)}^{0.75}\right].$$

To calculate the diffusion coefficient from the weight measurements, a fit of this approximate solution to the measured values can be done.

## 3. Results and Discussion

To analyze the moisture uptake of the CFRP plate, the relative mass change $M_{r}$ is calculated from the absolute values

(6) $$M_{r}=\frac{M\left(t\right)-M_{dry}}{M_{dry}}\;,$$

where M(t) is the mass at a specific time and $M_{dry}$ is the mass of the dried sample. 

**Figure 4:**
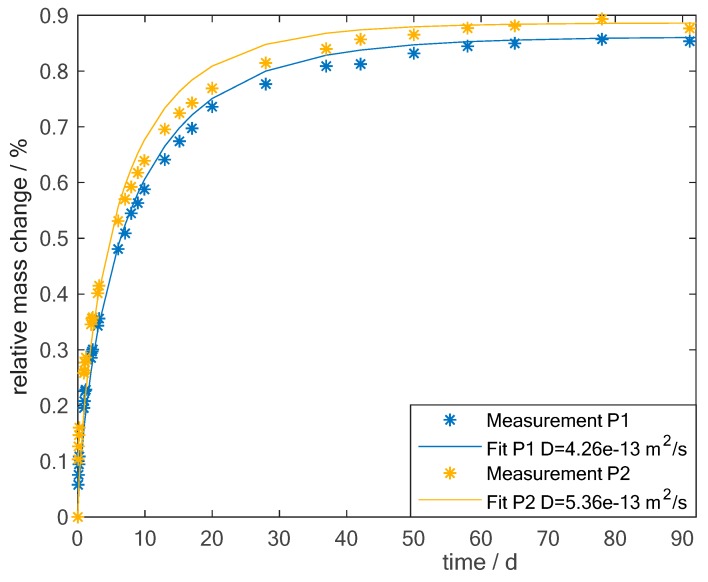
Mass change of two CFRP samples (see Table 2) with resin M21 during exposure to a hot-wet environment of 70°C and 85% relative humidity.

In Figure 4, the relative mass change is shown over time. The measurement procedure is described in Section 2.3. The stars indicate the measured values of the two samples P1 and P2 (see Table 2), while the lines are the corresponding fitted solution of Fick’s law, see Equation (5). Equation (5) represents the specific form of Fick’s law which can be applied here (see Section 2.4). The diffusion coefficient is not known exactly beforehand and is, therefore being used as the fitting parameter. The resulting diffusion coefficients are noted in the legend. The fit was done using a nonlinear least square technique, the trust region algorithm, implemented in Matlab. It can be seen that there are slight differences from the Fickian behaviour. The first reason may be the size of the samples, they are not infinitely large compared to the thickness and, therefore, diffusion may also appear through the edges. In addition, the fibers are not considered here in this approach. There are models which also consider the fibers and how they change the diffusive path [33]. However, as the model in general fits and the focus here is not the analysis of the diffusion process itself but the measurement with the material integrated sensors, these detailed models are not considered here. Moreover, microcracks and voids are always present in the matrix [11]. These also influence the moisture uptake and are not considered in the Fickian model. Canal published a diffusion coefficient of $1\times 10^{-13}\frac{m^{2}}{s}$ and a maximum amount of moisture of 1.3% for a 1.1 mm thick plate from M21 resin and T700 fibers immersed in water at 70°C [34]. This value of the diffusion coefficient is in the same range as the values measured here, considering the differences in experimental design. The maximum amount of water inside the material is correlated to the moisture of the surrounding material. Therefore, it is expected that the maximum amount of moisture uptake is higher for a sample immersed in water compared to a sample exposed to 85 % humidity. In the next step, the measured admittance values of the sensors are analyzed.

**Figure 5:**
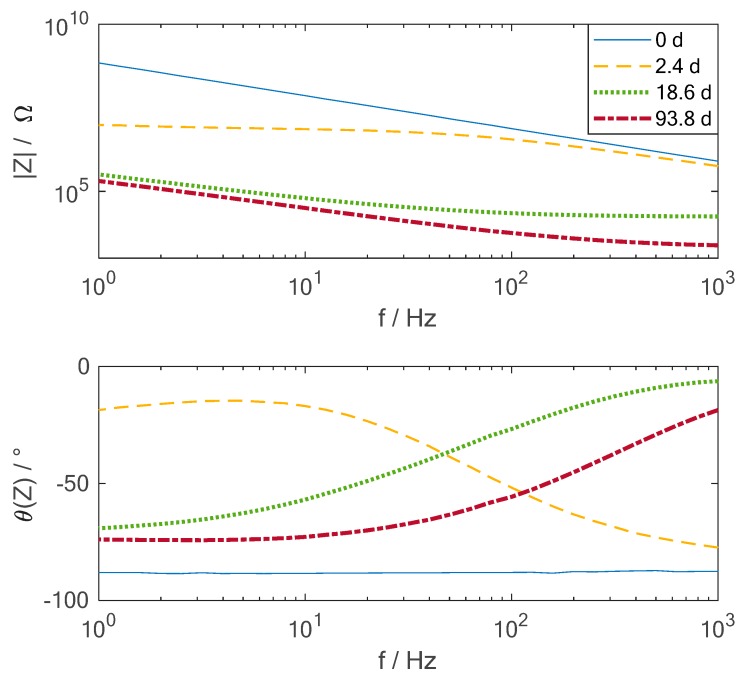
Amplitude and phase over frequency of the impedance of the interdigital sensors S2 integrated into the CFRP during exposure to a hot-wet environment of 70°C and 85% relative humidity for different times.

In Figure 5 the amplitude and phase of the impedance of one of the integrated sensors (S2) are shown over frequency for different times during the exposure of the CFRP sample to hot-wet conditions. The time spots chosen here for the graphic are just representatives for a certain period of the measurement, many more time measurements were recorded. At the beginning of the measurement, the sensor shows a clearly capacitive behaviour, the phase is nearly constant at 90° and the amplitude is a straight line in the double logarithmic representation. This is expected from theory as the resin itself does not contain any movable charges at this point in time. After 2.4 days the first impact from the water uptake can be observed. At frequencies below 50 Hz, the phase shifts towards zero and the amplitude becomes nearly frequency independent. This indicates a resistive behaviour in this specific frequency range due to relatively large particles which can transfer charges. This results from the diffusion of the water from the environment inside the material. The diffusion of the water molecules enables a movement of the charges inside the material at the beginning of the process. Over time, the amplitude drops due to the rising amount of water inside the material. The higher the content of the water, the lower are the impedance values. This can be expected from theory as the permittivity of water is higher than the one of the resin, which results in a lower impedance. To compare the electrical measurements with the sensors to the mass change of the samples during the hot-wet conditioning test, the impedance should be analyzed over time. Therefore, the frequency of 31.6 Hz is chosen, as it is located in the middle of the spectra and in addition has very low noise since at this frequency no coupling from other machines in the lab can be observed. Furthermore, not the impedance but the inverse, the admittance is chosen in the graphical representation as it increases during moisture uptake. 

**Figure 6:**
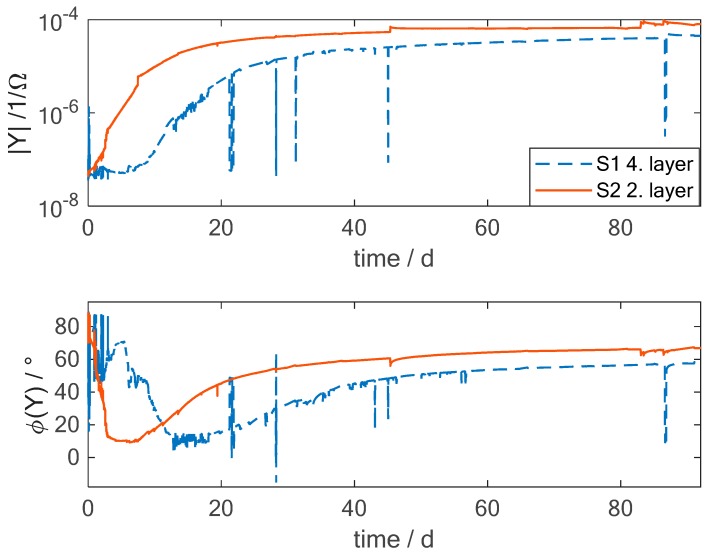
Admittance changes of two interdigital sensors integrated into the CFRP at a different position during exposure to a hot-wet environment of 70 °C and 85 % relative humidity. Here the measurement at a frequency of 31.6 Hz is shown. Sensor S1 is placed in the middle of the plate and S2 at ¼ of the plate.

**Figure 7:**
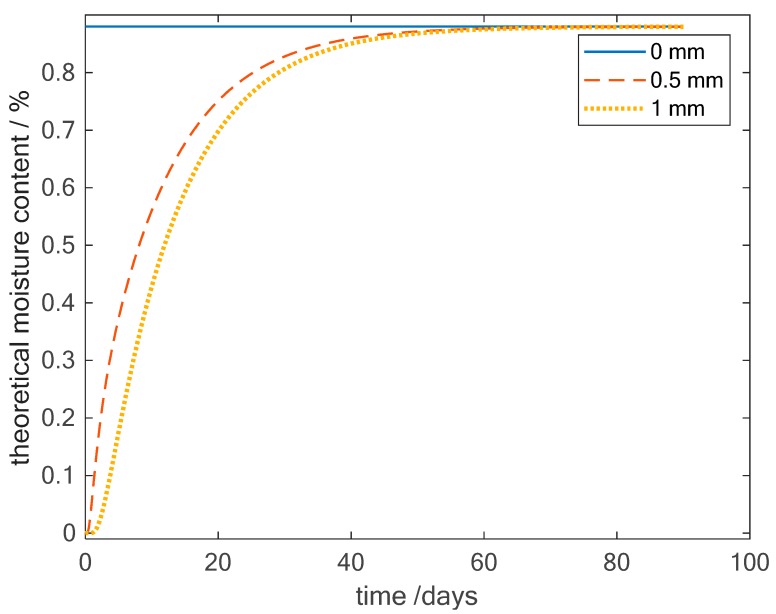
Theoretical mass change at the positions of the sensors S1 (1 mm) and S2 (0.5 mm).

In Figure 6, the amplitude and phase of the admittance of two material-integrated sensors are shown over time at a frequency of 31.6 Hz. The sensors are located at two different positions in the material. Sensor S1 is placed between layers 4 and 5; this means that it is located in the middle of the plate while sensor S2 is placed between layers 2 and 3, resulting in a position at one-fourth of the total thickness. Looking at the amplitude and phase, the two sensors show the same changes in the curves. The admittance rises quickly at the beginning and slower near the time when saturation is reached. This behaviour seems similar to the mass gain curves. The very narrow peaks measured by sensor S1 are a result of external distortions at the connection between the measurement device and the wires of the sensors and, therefore, can be neglected in the further analysis. The curves of the sensors seem to be shifted in time. Sensor S2 shows an increasing amplitude of the admittance already from the beginning while sensor S1 just reacts after around 8 days. As the two sensors are placed at a distance of 0.5 mm to each other, this seems to be a time shift which is quite high. Therefore Equation 3 is used to calculate the moisture mass change at the position of the two sensors. In Figure 7, the theoretical mass change curves at the positions of the sensors are shown. It can be seen that the changes in the middle of the plate (1 mm) are shifted in time compared to the values at the position of sensor S2 (0.5 mm). At the beginning of the measurement no moisture is assumed inside the material. On the outside of the sample the maximum moisture is present. Diffusion in the material starts from the outside and at the position 0.5 mm the moisture content increases first. In the course of time, the moisture reaches also the middle position at 1 mm. However, the time shift that can be expected from the theoretical value is around 1.5 days. This results in a large difference between the theoretical value at the middle of the plate and the measured values with the material integrated sensor. One possible explanation is the inhomogeneity of the CFRP. The theoretical equations fit for a homogeneous material and it was also demonstrated that the overall mass change can be represented by this model quite well. However, the specific behavior at different spots in the material may differ. For example, if the local fiber density above the sensor is very high compared to the overall material, then the diffusion at this point may be much slower. 

**Figure 8:**
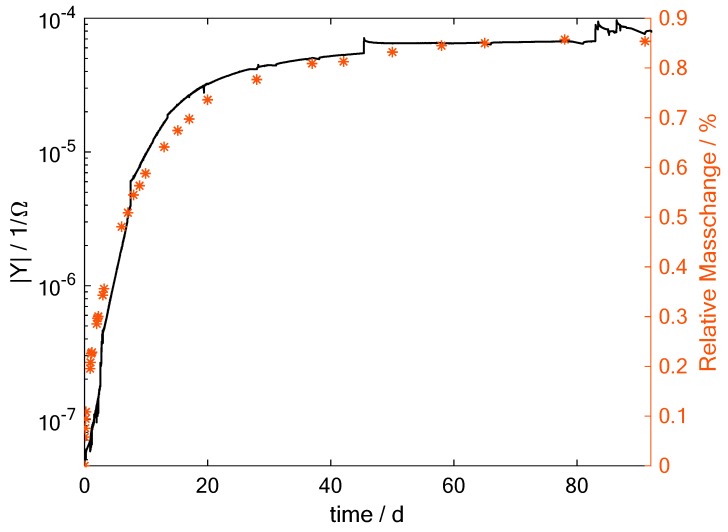
Amplitude of admittance changes Sensor S2 compared to the mass change during hot-wet test. Here the measurement at a frequency of 31.6 Hz is shown.

In the next step, the amplitude of the admittance of sensor S2 is directly compared to the mass change measured. In Figure 8 the amplitude of the admittance is shown in black and the measured mass change values in red stars. It can be observed that the admittance curve seems to fit the mass change quite well. Therefore, the sensors can be used to determine the actual moisture uptake in the CFRP part locally when the saturation moisture uptake and the diffusion coefficient are known. 

To use the sensors for direct moisture measurement the admittance moisture relation has to be recorded for each specific material. Then the sensors could be used in fabricated CFRP parts. The relative amount of moisture can be calculated from the following equation

(7) $$M_{r}=a\left(f\right)log\left(\left|Y\right|\right)+b\left(f\right)\;,$$

where *a* and *b* are frequency and material dependent coefficients and |Y| is the amplitude of the admittance. For the |*Y*| at a frequency of 31.6 Hz shown in Figure 8, the value for *a* is calculated as $a\left(31,6\;\mathrm{Hz}\right)=2.14\times 10^{-3}$ and $b\left(31,6\;\mathrm{Hz}\right)=17.6\times 10^{-3}$. With these coefficients once determined for a specific material, the relative moisture content can be calculated. 

## 4. Conclusions

A material integrated sensor in carbon fiber composite is presented. The sensor is highly flexible and thin, which makes it suitable to be integrated into the material without harming it. The thickness of the sensor is in the range of the diameter of the fiber. The interdigital sensor is used for monitoring the curing process during the fabrication of a CFRP part by measuring the impedance. Here, it is demonstrated that the already integrated sensor is also suitable for structural health monitoring during the service life of a CFRP product. It was shown that it is possible to measure the local amount of moisture in the CFRP using the integrated sensor. In a first instance, parameters like saturation, the amount of moisture and diffusion coefficient, were determined by measuring the mass changes during exposure to hot-wet conditions and fitting this measurement with the Fickian diffusion model. Comparing the mass change values with the measurements of the sensors, which were performed simultaneously, it can be shown that the admittance changes can be correlated to the mass changes. Therefore, the material integrated sensor is suitable to measure the moisture uptake inside the composite. If the saturation value and the diffusion coefficient are known, the moisture value can be directly identified from the admittance value. 

In general, it was demonstrated that the material integrated miniaturized flexible sensors, which were already successfully used for cure monitoring during the fabrication process of CFRP, can be used to monitor the moisture diffusion in CFRP online. Compared to traditional methods like gravimetric analysis or near infrared spectroscopy, the presented method can be used for online monitoring of CFRP parts during service time. This is possible because the sensor is very small and flexible compared to other sensors and does not influence the mechanical properties of the material. 

## Figures and Tables

**Table 1:** Dimensions of the used sensors.

Sensor	N	L	w	s
S1	300	3000 mm	10 µm	10 µm
S2	150	6000 mm	10 µm	10 µm

**Table 2:** Dimensions of the CFRP samples.

Sample	b	d	Sensor
P1	20 mm	100 mm	no sensor
P2	20 mm	100 mm	no sensor
M1	61 mm	100 mm	S1 between layer 4–5
M2	35 mm	110 mm	S2 between layer 2–3
